# Interleukin-12p35 Deficiency Reverses the Th1/Th2 Imbalance, Aggravates the Th17/Treg Imbalance, and Ameliorates Atherosclerosis in ApoE-/- Mice

**DOI:** 10.1155/2019/3152040

**Published:** 2019-04-10

**Authors:** Ying Huang, Haiying Hu, Ling Liu, Jing Ye, Zhen Wang, Bin Que, Wenjing Liu, Ying Shi, Tao Zeng, Lei Shi, Qingwei Ji, Chao Chang, Yingzhong Lin

**Affiliations:** ^1^Department of Cardiology, The People's Hospital of Guangxi Zhuang Autonomous Region, Nanning, China; ^2^Department of Ultrasound, The People's Hospital of Guangxi Zhuang Autonomous Region, Nanning 530021, China; ^3^Department of Cardiology, Handan First Hospital, Handan 056002, China; ^4^Department of Cardiology, Renmin Hospital of Wuhan University and Cardiovascular Research Institute, Wuhan University and Hubei Key Laboratory of Cardiology, Wuhan 430060, China; ^5^Emergency & Critical Care Center, Beijing Anzhen Hospital, Capital Medical University, and Beijing Institute of Heart, Lung, and Blood Vessel Diseases, Beijing 100029, China

## Abstract

Interleukin- (IL-) 35, a novel functional cytokine of regulatory T cells (Treg) comprised of the IL-12p35 subunit and the other subunit Epstein-Barr virus-induced gene 3 (EBI3), regulates the activity of CD4+ T cells and macrophages, thereby playing a critical role in inflammatory and autoimmune diseases. Previous studies demonstrated that both recombinant mice and human IL-35 attenuated atherosclerosis in ApoE-/- mice. Additionally, EBI3 deficiency enhanced the activation of macrophages and increased atherosclerotic lesions in LDLR-/- mice. This study generated double-deficient mice for ApoE and IL-12p35 (ApoE-/- IL-12p35-/- mice) and investigated the effect of IL-12p35 deficiency on atherosclerosis. IL-12p35 deficiency alleviated Th1/Th2 imbalance, aggravated Th17/Treg imbalance, and attenuated atherosclerotic plaque formation in ApoE-/- mice. Additionally, exogenous rIL-35 treatment reversed the imbalance of Th17/Treg and attenuated atherosclerosis in ApoE-/- mice. These findings suggest that IL-12p35 deficiency ameliorates atherosclerosis in ApoE-/- mice, partially, via attenuating the Th1/Th2 imbalance, although IL-12p35 deficiency aggravates the Th17/Treg imbalance.

## 1. Introduction

Atherosclerosis is a common clinical disease which is caused by multifactorial factors and leads to different degrees of vascular stenosis and blockage and clinical manifestations. Although the specific mechanisms of atherosclerosis remain unclear, disorders of inflammatory cells, including CD4+ T cells and macrophages, were considered to play a critical role in the progression of atherosclerosis [1–3].

CD4+ T cells include Th1, Th2, Th17, and regulatory T (Treg) cells. Th1, Th2, and Th17 participate in diseases mainly via promoting the expression of interferon- (IFN-) *γ*, interleukin- (IL-) 4, and IL-17. Accumulating evidence demonstrated that both Th1 and Th17 responses played a pathogenic role in atherosclerosis, while IFN-*γ* or IL-17 deficiency alone ameliorated atherosclerosis in ApoE-/- mice [4–7]. Although Th2 has a property against Th1 immune response, IL-4 deficiency attenuated atherosclerosis both in ApoE-/- and in LDLR-/- mice, suggesting an atherogenic role of IL-4 during atherosclerosis [8, 9]. However, other Th2-type cytokines such as IL-5, IL-13, and IL-33 were found to protect mice from atherosclerosis [10–14]. Taken together, these reports indicated a complex role of Th2 immune response in atherosclerosis. Treg cells, identified in the 1990s, were found to suppress Th1-, Th2-, and Th17-type immune responses and maintain the immune tolerance and immune homeostasis through cell-cell contact, elimination of effector T cell growth factors such as IL-2, and secretion of inhibitory cytokines such as IL-10 and transforming growth factor- (TGF-) *β* [15, 16]. Numerous studies have demonstrated a protective role of Treg cells in atherosclerosis [17–19].

IL-35 is a novel anti-inflammatory cytokine that belongs to the IL-12 family [20]. IL-35 is composed of an IL-12p35 subunit and an Epstein-Barr virus-induced gene 3 (EBI3) subunit. An IL-12p35 subunit binds with an IL-12p40 subunit to form IL-12 while an EBI3 subunit binds with an IL-27p28 subunit to form IL-27. IL-35 was found to play a critical role in Treg-mediated immune homeostasis maintenance and has been considered a functional cytokine of Treg [20–22]. Using immunocytochemical staining methods, Kempe et al. found that IL-12p35 is strongly expressed in advanced human carotid plaque samples with EBI3 [23]. We found that circulating IL-35 concentrations significantly decreased in patients with coronary artery disease [24]. This evidence indicated that IL-35 is involved in the regulation of human atherosclerosis. More recently, Tao et al. found that both exogenous recombinant human and mouse IL-35 treatments reduced atherosclerosis in ApoE-/- mice, suggesting an atheroprotective effect of IL-35 [25, 26]. Additionally, EBI3, the *β* chain of IL-35 and IL-27 deficiency, enhanced the accumulation and activation of macrophages and aggravated the development of atherosclerosis in LDLR-/- mice [27]. This study generated double-deficient mice for ApoE and IL-12p35 (ApoE-/- IL-12p35-/- mice) and investigated the role of IL-12p35 deficiency in atherosclerosis.

## 2. Materials and Methods

### 2.1. Animals and Animal Models

Both the ApoE-/- mice and IL-12p35-/- mice (both from Jackson Laboratory, USA) were used in this study. The ApoE-/- IL-12p35-/- mice were generated after the ApoE-/-, and IL-12p35-/- mice were crossbred for three generations. All the mice were housed in a specific pathogen-free mouse room in Renmin Hospital of Wuhan University, and male mice aged 8 weeks were used in this study. Both the ApoE-/- mice (*n* = 16) and ApoE-/- IL-12p35-/- mice (*n* = 16) were fed a high-fat diet (HFD) which includes 21% fat and 0.15% cholesterol for 24 weeks. The weight of each mouse was measured weekly at the same time, and the blood samples were collected from the angular vein after these mice were fed for 8 weeks, 18 weeks, and 24 weeks, respectively. In addition, other ApoE-/- mice were intraperitoneally injected (ip) daily with phosphate-buffered saline (PBS, 50 *μ*l, *n* = 16) and rIL-35 (0.75 *μ*g, purity ≥ 98%, PeproTech, *n* = 16) [18], respectively. At the end of the 24th week, all mice were then euthanized and the blood samples, spleen, hearts, and aortas were collected for further analysis. This study was reviewed and approved by the Institutional Animal Care and Use Committee at the People's Hospital of the Guangxi Zhuang Autonomous Region (Nanning, China) and Renmin Hospital of Wuhan University (Wuhan, China) and conducted in accordance with the guidelines from the directive 2010/63/EU of the European Parliament.

### 2.2. Lipid Measurement

Serum was separated from the blood samples which were collected at the 8th week, 18th week, and 24th week. The concentrations of total cholesterol (TC), triglycerides (TG), high-density lipoprotein cholesterol (HDL-C), and low-density lipoprotein cholesterol (LDL-C) in each sample were measured by enzymatic methods using kits from BioVision (Mountain View, UAS).

### 2.3. Oil Red O Staining

The aortic tree was opened longitudinally and then stained with 5% oil red O (Sigma, USA) for 30 minutes. After being washed in running distilled water for 3 times (15 s/time), the aortas were pinned down and photographed using a digital camera to acquire images of the en face lesion. In addition, the hearts were fixed in paraformaldehyde for 120 minutes and placed in a solution of 30% sucrose in PBS overnight at 4°C. Then, the hearts were embedded in an optimum cutting temperature (OCT) compound and frozen at -80°C. The hearts containing the aortic root were cut into approximately 4-5 *μ*m sections; the oil red O staining of each sample and washing were performed as the description above.

### 2.4. Immunohistochemical Analysis of Lesion

Approximately 4-5 *μ*m sections of the aortic sinus were prepared. Then, the sections were incubated overnight at 4°C with the primary antibodies anti-*α* smooth muscle actin (*α*-SMA) (Abcam, USA), anti-CD4 (R&D Systems, USA), and anti-CD68 (R&D Systems) in order to detect the expression of smooth muscle cells, CD4+ T lymphocyte, and macrophages, respectively. Masson trichrome was performed to measure the collagen expression in the plaque area.

### 2.5. Analysis of CD4+ T Helper Cells

The mouse spleens were grinded and used to prepare single cell suspension; then, these cells were put into an RPMI 1640 (Gibco, UAS) complete culture medium. The total T lymphocytes were extracted from the spleen single cell suspension using lymphocyte separation liquid (Sigma, UAS). After being resuspended at a density of approximately 5 × 10^6^ cells/ml in RPMI 1640 medium, the T lymphocytes were stimulated with 2 *μ*/ml cell stimulation cocktail (eBioscience, UAS) in an environment with 5% CO_2_ at 37°C for 5 hours. Then, the cells were collected and treated with phycoerythrin Cye-7-conjugated anti-mouse CD4 (PE-Cy7-CD4) for 30 minutes. After being treated with fixation/permeabilization concentrate at room temperature for 60 min, the total T lymphocytes were further stained with phycoerythrin- (PE-) labeled anti-IFN-*γ* (PE-IFN-*γ*), PE-labeled anti-IL-4 (PE-IL-4), PE-labeled anti-IL-17 (PE-IL-17), or PE-labeled anti-CD25 (PE-CD25)+APC-labeled anti-Foxp3 (APC-Foxp3) for 30 minutes. Isotype controls were included for compensation and to confirm antibody specificity. Th1 cells were defined as CD4+IFN-*γ*+, Th2 cells were defined as CD4+IL-4+, Th17 cells were defined as CD4+IL-17+, and Treg cells were defined as CD4+CD25+Foxp3. All of the flow cytometry antibodies were purchased from eBioscience and used according to the manufacturer's instructions.

### 2.6. Cytokine Assay

The serum IFN-*γ*, IL-4, IL-17, and IL-10 levels after mice were fed a HFD for 24 weeks were measured using enzyme-linked immunosorbent assay (ELISA, all from eBioscience) kits according to the manufacturer's instructions.

### 2.7. Analysis of mRNA Expression

After being treated with TRIZOL Reagent W, the total mRNA in the aortas and spleens was extracted. After the concentration of each sample was measured, a total of 2 *μ*g mRNA and oligo (dT) primers were used to synthesize the cDNA by a reverse transcription kit according to the manufacturer's instructions. Then, the LightCycler 480 SYBR Green Master Mix (all from Roche) was used for the amplifications of PCR. The relative mRNA expression levels of ROR*γ*T, IL-17, IL-6, TNF-*α*, Foxp3, IL-10, IFN-*γ*, GATA3, and IL-4 were measured, and the results were normalized against the expression levels of GAPDH. The RT-qPCR primer sequences are shown in Table 1.

### 2.8. Statistical Analysis

All data are expressed as the mean ± standard deviation (SD) and were analyzed by SPSS 22 software. The differences between the two groups were compared with unpaired Student's *t*-test. A *P* value < 0.05 was considered statistically significant.

## 3. Results

### 3.1. IL-12p35 Deficiency Alleviated Atherosclerosis in ApoE-/- Mice

**Figure 1:**
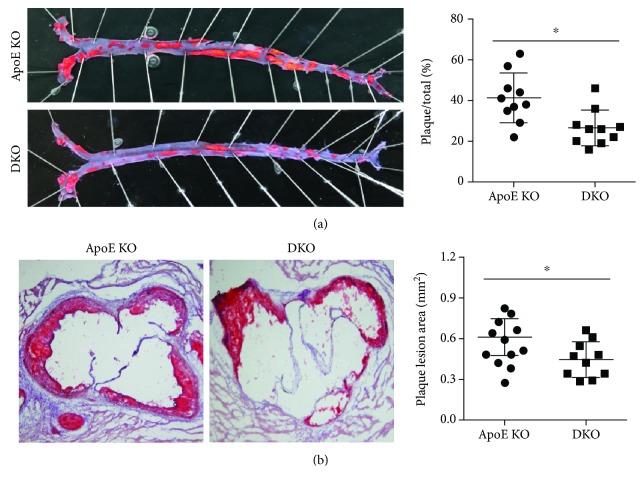
The atherosclerotic plaque area of the ApoE-/- mouse and ApoE-/- IL-12p35-/- mouse groups. The atherosclerotic plaque area of (a) aortic trees (10x) and (b) aortic roots (10x) in these two groups was detected by oil red O staining. ApoE KO = ApoE-/-, DKO = ApoE-/- IL-12p35-/-; *N* = 10 for each group. ^∗^*P* < 0.05.

The atherosclerotic plaque of each mouse by oil red O straining and the results showed that in HDF-fed ApoE-/- mice, IL-12p35 deficiency significantly reduced the atherosclerotic plaque approximately 35% in the aortic trees (ApoE-/- group: 41 ± 12% vs. ApoE-/- IL-12p35-/- group: 27 ± 9%, Figure 1(a)), while exhibiting a reduction of approximately 26% atherosclerotic plaque in the aortic root (ApoE-/- mouse group: 0.61 ± 0.14 mm^2^ vs. ApoE-/- IL-12p35-/- mouse group: 0.44 ± 0.13 mm^2^, Figure 1(b)).

### 3.2. Effect of IL-12p35 Deficiency on Serum Lipid Levels and Body Weight

The serum lipid levels and body weight in different time points after the mice were fed a HDF were measured; the results showed that no difference of TC, TG, HDL-C, and LDL-C levels and body weight were observed in weeks 8, 18, and 24. The serum lipid levels and body weight of each group in different time points are listed as Table 2.

### 3.3. Effect of IL-12p35 Deficiency on the Formation of Atherosclerotic Plaques

**Figure 2:**
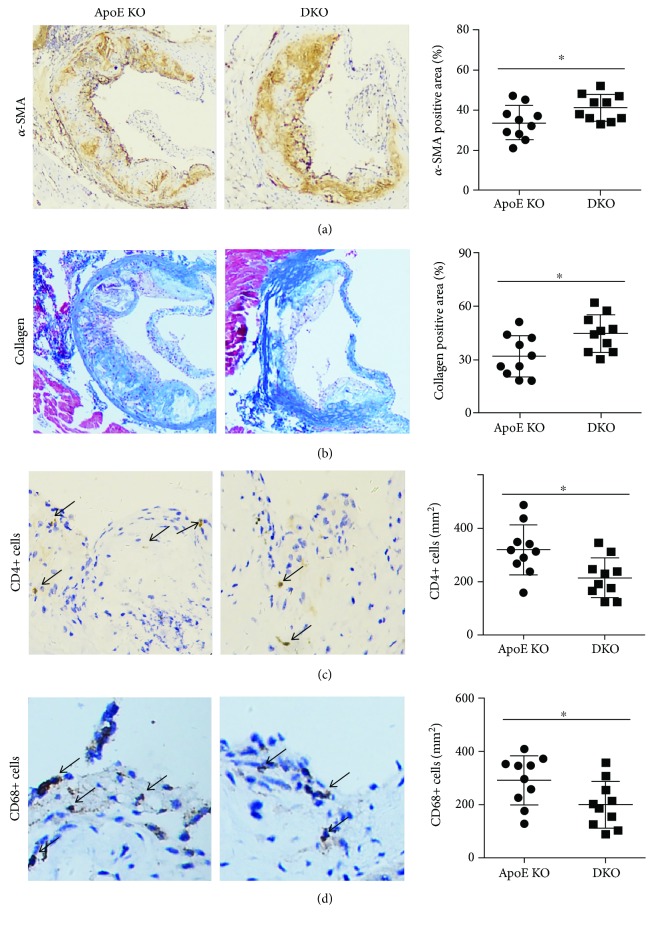
Effect of IL-12p35 deficiency on the stability of atherosclerotic plaque in the aortic sections of ApoE-/- mice. The aortic roots of (a) *α*-SMA-positive area (20x), (b) collagen-positive area (20x), (c) CD4+ T lymphocyte (40x), and (d) macrophages (40x) were measured in the two groups. ApoE KO = ApoE-/-; DKO = ApoE-/- IL-12p35-/-; *N* = 10 for each group. ^∗^*P* < 0.05.

As the results of immunohistochemical analysis, the *α*-SMA-positive areas of the aortic root were significantly increased by IL-12p35 deficiency (Figure 2(a)); in addition, higher collagen expression was found in the aortic root of ApoE-/- IL-12p35-/- mice when compared with that of ApoE-/- mice (Figure 2(b)); furthermore, the infiltration of both CD4+ T lymphocyte and macrophages in the aortic root was significantly decreased in ApoE-/- IL-12p35-/- mice (Figures 2(c) and 2(d)).

### 3.4. IL-12p35 Deficiency Modified the Activity of CD4+ T Cells in ApoE-/- Mice

**Figure 3:**
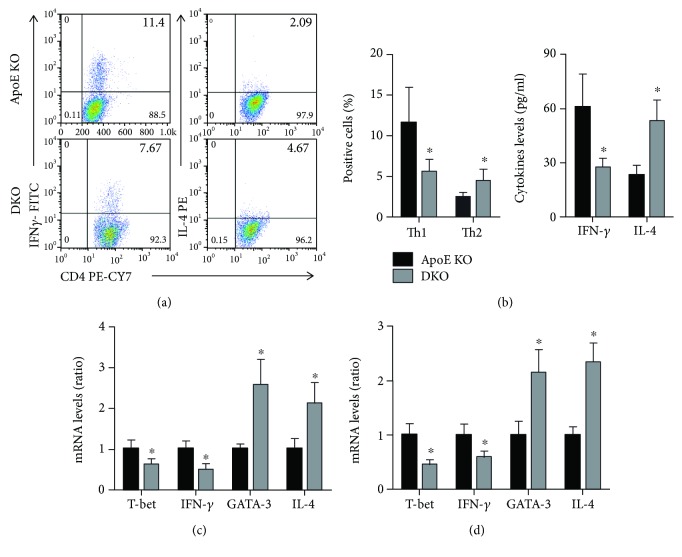
Effect of IL-12p35 deficiency on the activity of Th1 and Th2. (a) The frequency of Th1 and Th2 in ApoE-/- mice and ApoE-/- IL-12p35-/- mice was measured by flow cytometry, *n* = 10 for each group. (b) The serum IFN-*γ* and IL-4 levels were detected by ELISA; *n* = 16 for each group. (c, d) The mRNA levels of T-bet, IFN-*γ*, GATA3, and IL-4 in the spleens and aortas of these two groups were analyzed by RT-qPCR. ApoE KO = ApoE-/-, DKO = ApoE-/- IL-12p35-/-; *N* = 6 for each group. ^∗^*P* < 0.05 vs. the ApoE-/- group.

The percentages of Th1 cells and Th2 cells in the spleen were detected by flow cytometry. The results showed that IL-12p35 deficiency decreased Th1 percentages but increased Th2 percentages (Figure 3(a)). In addition, the serum IFN-*γ* and IL-4 levels were measured by ELISA and lower IFN-*γ* levels and higher IL-4 levels were observed in the ApoE-/- IL-12p35-/- mouse group when compared with the ApoE-/- group (Figure 3(b)). Furthermore, the transcription factors and functional cytokines of mRNA levels of Th1 and Th2, including T-bet, IFN-*γ*, GATA3, and IL-4, were analyzed by RT-qPCR and the reduction trend of T-bet and IFN-*γ* mRNA levels as well as the increase trend of GATA3 and IL-4 mRNA levels was found in both the spleens and aortas of ApoE-/- IL-12p35-/- mice compared with ApoE-/- mice (Figures 3(c) and 3(d)).

**Figure 4:**
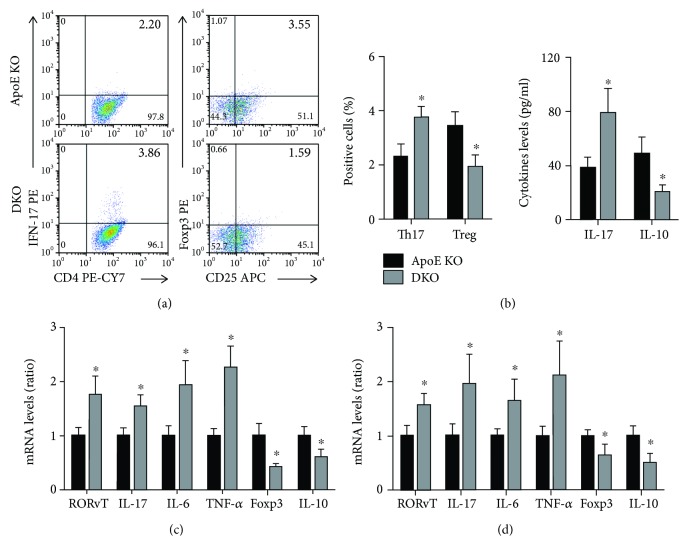
Effect of IL-12p35 deficiency on the activity of Th17 and Treg. (a) The frequency of Th17 and Treg in ApoE-/- mice and ApoE-/- IL-12p35-/- mice was measured by flow cytometry; *n* = 10 for each group. (b) The serum IL-17 and IL-10 levels were detected by ELISA; *n* = 16 for each group. (c, d) The mRNA levels of ROR*γ*T, IL-17, IL-6, TNF-*α*, Foxp3, and IL-10 in the spleens and aortas of these two groups were analyzed by RT-qPCR. ApoE KO = ApoE-/-, DKO = ApoE-/- IL-12p35-/-; *N* = 6 for each group. ^∗^*P* < 0.05 vs. the ApoE-/-group.

Th17 levels were increased, and Treg levels were reduced in ApoE-/- IL-12p35-/- mice (Figure 4(a)). IL-12p35 deficiency also exhibited an increase in serum IL-17 levels and a reduction in IL-10 levels (Figure 4(b)). In addition, lower ROR*γ*T, IL-17, IL-6, and TNF-*α* mRNA and higher Foxp3 and IL-10 mRNA expression were observed in both the spleen and aortas of ApoE-/- IL-12p35-/- mice when compared with ApoE-/- mice (Figures 4(c) and 4(d)).

### 3.5. Exogenous Mouse rIL-35 Treatment Reduced Atherosclerosis in ApoE-/- Mice

**Figure 5:**
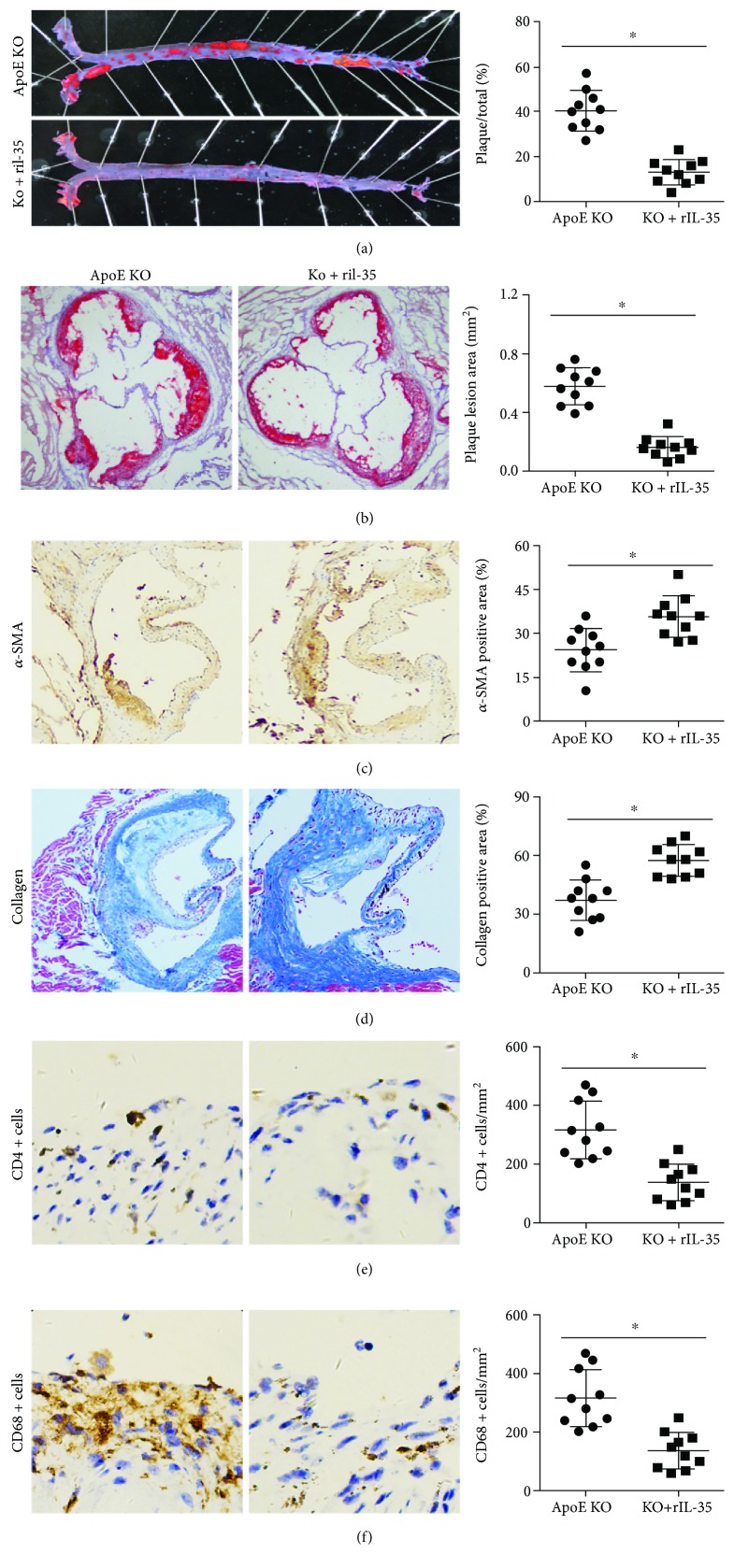
rIL-35 ameliorates the development of atherosclerosis. The atherosclerotic plaque area of (a) aortic trees (10x) and (b) aortic roots (10x) in these two groups was detected by oil red O staining. The aortic roots of (c) *α*-SMA-positive area (20x), (d) collagen-positive area (20x), (e) CD4+ T lymphocyte (40x), and (f) macrophages (40x) were measured in the two groups. ApoE KO = ApoE-/-, DKO = ApoE-/- IL-12p35-/-; *N* = 10 for each group. ^∗^*P* < 0.05 vs. the ApoE-/- group.

After the ApoE-/- mice were given rIL-35, we firstly detected the serum lipid levels and body weight in weeks 8, 18, and 24 and the results showed that rIL-35 did not affect serum lipid levels and body weight in ApoE-/- mice; the serum lipid levels and body weight in different time points are listed in Table 3. Treatment with rIL-35 significantly reduced approximately 68% of the atherosclerotic plaque area (ApoE-/- group: 40 ± 9% vs. rIL-35 group: 13 ± 6%, Figure 5(a)) in the aortic tree and approximately 72% of the atherosclerotic plaque area (ApoE-/- group: 0.57 ± 0.12 mm^2^ vs. rIL-35 group: 0.16 ± 0.07 mm^2^, Figure 5(b)) in the aortic root. In addition, the *α*-SMA and collagen expression was increased while the infiltration of T lymphocyte and macrophages was decreased in the rIL-35 treatment group (Figures 5(c)–5(f)).

### 3.6. Exogenous Mouse rIL-35 Treatment Modulated the Activity of CD4+ T Cells

**Figure 6:**
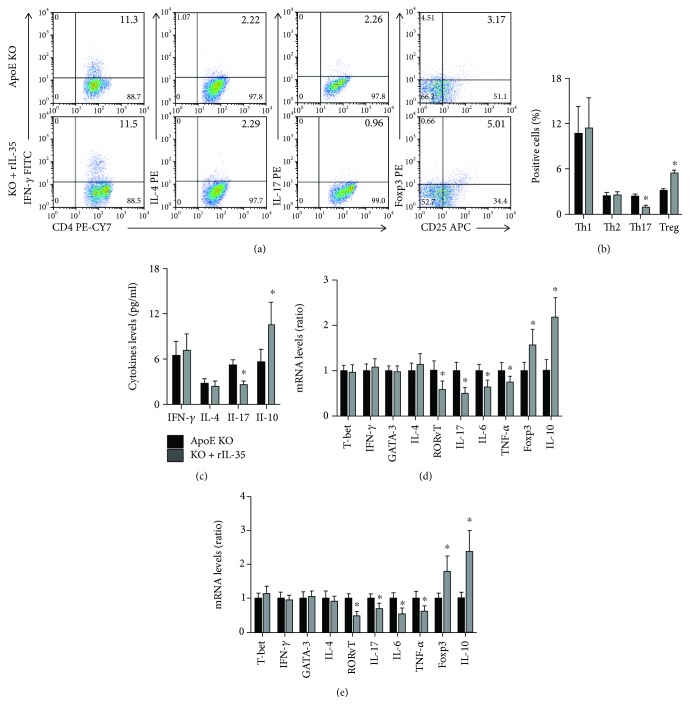
Effect of rIL-35 on the activity of CD4+ T cells. (a, b) The frequency of Th1, Th2, Th17, and Treg in the ApoE-/- and ApoE-/-+rIL-35 groups was measured by flow cytometry; *n* = 10 for each group. (c) The serum IFN-*γ*, IL-4, IL-17, and IL-10 levels were detected by ELISA; *n* = 16 for each group. (d, e) The mRNA levels of T-bet, IFN-*γ*, GATA3, IL-4, ROR*γ*T, IL-17, IL-6, TNF-*α*, Foxp3, and IL-10 in the spleens and aortas of these two groups were analyzed by RT-qPCR. ApoE KO = ApoE-/-, DKO = ApoE-/- IL-12p35-/-; *N* = 6 for each group. ^∗^*P* < 0.05 vs. the ApoE-/- group.

The effect of rIL-35 treatment on the activity of CD4 T lymphocyte was measured. The results exhibited that rIL-35 did not affect Th1 and Th2 levels, while preventing Th17 levels and promoting Treg levels in ApoE-/- mice (Figures 6(a) and 6(b)). Exogenous mouse rIL-35 treatment also had no effect on IFN-*γ* and IL-4 levels, while decreasing IL-17 levels and increasing IL-10 levels (Figure 6(c)). In addition, no differences of the transcription factors and functional cytokine mRNA levels of Th1 cells and Th2 cells in both the spleens and aortas were found between the ApoE-/- and rIL-35 groups; the Th17-related transcription factors and functional cytokine mRNA were decreased, and the Treg-related transcription factors and functional cytokine mRNA were increased (Figures 6(d) and 6(e)).

## 4. Discussions

The IL-12 cytokine family includes four members: IL-12, IL-23, IL-27, and IL-35, which are heterodimer proteins that consist of an *α* subunit (p19, p28 or p35) and a *β* subunit (EBI3 or p40) [28]. IL-12 is composed of p35 and p40 subunits, IL-23 is composed of p40 and p19 subunits, IL-27 is composed of EBI3 and p28, and IL-35 is composed of p35 and EBI3 subunits. Both IL-12 and IL-27 play a critical role in the induction of Th1 cells, whereas IL-23 is critical for inducing a pathogenic phenotype in Th17 cells. Among the IL-12 cytokine family, only IL-35 is mainly secreted by Treg cells, in turn inducing Treg cells and maximizing their suppressive capacity.

Over the last couple of decades, atherosclerosis has been considered a CD4+ T cell-mediated chronic inflammatory disease. CD4+ T cells were the mostly wildly studied during atherosclerosis and predominant both in human and in mouse atherosclerotic lesions [2, 3]. The imbalance of Th1/Th2 and the imbalance of Th17/Treg played a pathogenic role during the development of atherosclerosis [4–7, 10, 17–19] and were associated with the onset of acute coronary syndrome [29, 30], even unfavorable prognosis in patients with atherosclerotic diseases [31–33]. Additionally, numerous studies proved that reversing the imbalance of Th1/Th2 or the imbalance of Th17/Treg significantly attenuated atherosclerosis [2, 10, 24, 34, 35]. Accumulating evidence demonstrated that the IL-12 cytokine family plays a vital role in atherosclerosis through regulating the activity of CD4+ T cells. Using IL-12p40-deficiency mice or recombinant IL-12, previous studies have proved the atherogenic role of IL-12 [9]. IL-23 treatment promoted Th17 response and macrophage apoptosis, thereby resulting in an unstable plaque phenotype in LDLR-/- mice, although it had no effect on the lesion size [36]. Therefore, IL-23 may play adverse effects in atherosclerosis. The augment effect of IL-27 using recombinant IL-27 inhibited macrophage activation and attenuated atherosclerosis, while blockade of IL-27/IL-27R using EBI3-deficiency mice and transplanting IL-27R*α*−/− bone marrow to LDLR-/- mice induced the Th17 immune response and aggravated atherosclerosis, indicating a protective role of IL-27 in atherosclerosis [27, 37]. Because EBI3 is shared by IL-27 and IL-35, EBI3 deficiency means the combined effect of IL-27 and IL-35 deficiency.

In the present study, we used IL-12p35-/- mice and ApoE-/- mice to generate ApoE-/- IL-12p35-/- mice to investigate whether the *α* subunit of IL-35 has effects on atherosclerosis and to explore its potential mechanisms. The results showed that IL-12p35 deficiency decreased the infiltration of CD4+ T cells and macrophages and increased vascular smooth muscle cells and collagen in the plaques of the ApoE-/- mice, suggesting that IL-12p35 deficiency not only attenuates atherosclerosis but also elicits a stable plaque phenotype, thereby playing a protective role in atherosclerosis. To explore the mechanisms of IL-12p35 deficiency involved in atherosclerosis, the frequency of spleen Th1, Th2, Th17, and Treg cells was detected in the present study. Interestingly, IL-12p35 deficiency alleviated Th1/Th2 imbalance, which means IL-12 deficiency, but aggravated Th17/Treg imbalance, which means IL-35 deficiency. We speculated that the overall effect of IL-12p35 deficiency on atherosclerosis depends on the dominant position between the alleviated Th1/Th2 imbalance and the aggravated Th17/Treg imbalance induced by IL-12p35 deficiency.

Previous studies had demonstrated that IL-12p35 deficiency was closely related to cardiovascular diseases. In an earlier study, Fairweather et al. reported that IL-12p35 deficiency reduced the expression of IFN-*γ*, macrophage, and neutrophil populations in the heart and protected against coxsackievirus B3-induced myocarditis [38]. In another study, Li et al. reported that IL-12p35 deficiency enhanced angiotensin II-induced cardiac fibrosis via promoted CD4+ T helper cell-dependent M2 macrophage differentiation and upregulated M2 macrophage differentiation-related transformation growth factor *β*1 activation [39]. In a later study, Kan et al. found that IL-12p35 deficiency protected against chronic myocardial infarction which is mediated by the ligation of left anterior descending branch via promoting angiogenesis and reduced inflammatory response [40]. In the present study, we found that IL-12p35 deficiency suppressed inflammation and relieved the atherosclerotic area in both the aortic trees and aortic roots, suggesting the involvement of IL-12p35 deficiency in atherosclerosis. Taken together, these results indicated that IL-12p35 deficiency plays an important role in the regulation of inflammation.

In the present study, we also used recombinant mouse IL-35 to treat ApoE-/- mice to further strengthen the role of IL-12p35 in atherosclerosis. The results showed that rIL-35 treatment inhibited Th17 immune response, upregulated Treg response, and reduced atherosclerosis in ApoE-/- mice. However, the imbalance of Th1/Th2 was not reversed by rIL-35 treatment. Therefore, IL-12p35 deficiency means the combined effect of IL-12 and IL-35 deficiencies, which resulted in an inhibition in Th1 and Treg immune responses, whereas rIL-35 only affects the imbalance of Th17/Treg. Recently, Tao et al. found that treatment with recombinant human IL-35 led to an increase in both circulating and local Treg levels and a reduction in the plaque size in ApoE-/- mice, suggesting that IL-35 attenuates atherosclerosis via upregulating Treg immune response. Additionally, Li et al. found that IL-35 was upregulated in the atherosclerotic plaque in ApoE-/- mice and recombinant mouse IL-35 reduced atherosclerosis via suppressing endothelial cell activation [26]. Taken together, these results indicated an antiatherosclerotic role of IL-35, at least partly, through inhibiting inflammation.

Hyperlipidemia is one of the most important reasons of atherosclerosis, because elevated TC and LDL-C could lead to severe inflammatory response [41], which was closely related to the atherosclerosis department. In addition, hyperlipidemia also could cause the damage of vascular endothelial function, while the endothelial dysfunction is the initiating factor for cardiovascular disease, including hypertension, aortic dissection, and atherosclerosis. A previous study found that recombinant human IL-35 reduced the serum levels of TC and TG, but not affected the LDL-C and HDL-C levels in ApoE-/- mice [25], indicating that the effect of IL-35 on lowering lipids is associated with the reduction of atherosclerosis. Therefore, we measured the dynamic serum lipid levels to confirm whether IL-12p35 is involved in the process of atherosclerosis by regulating blood lipid levels. The results showed that IL-12p35 deficiency did not affect the serum lipid levels in HDF-fed ApoE-/- mice. Additionally, recombinant mouse IL-35 also had no obvious effect on blood lipid levels. These results suggest that the antiatherosclerotic role of IL-12p35 deficiency is independent on the regulation of blood lipids. The reason for our inconsistency with previous conclusions may be that the species from which IL-35 is derived is different.

In conclusion, we found that IL-12p35 deficiency alleviated atherosclerosis in ApoE-/- mice; the antiatherosclerotic effect of IL-12p35 deficiency was verified by the regulation of inflammation and was the result of the interaction between IL-12 and IL-35. On the other hand, exogenous IL-35 reduced atherosclerosis, at least in part, by modifying the imbalance of Th17/Treg. The present study indicated that IL-12p35 is a novel prevention and treatment target for atherosclerosis.

## Figures and Tables

**Table 1:** RT-qPCR primers used.

Gene	Forward primer	Forward primer
T-bet	CAACAACCCCTTTGCCAAAG	TCCCCCAAGCAGTTGACAGT
IFN-*γ*	GCTGTTACTGCCACGGCACA	GGACCACTCGGATGAGCTCA
GATA3	TCTGGAGGAGGAACGCTAATGG	GAACTCTTCGCACACTTGGAGACTC
IL-4	CCTCACAGCAACGAAGAACA	ATCGAAAAGCCCGAAAGAGT
ROR*γ*T	CGCCTCACCTGACCTACC	TTGCCTCGTTCTGGACTATAC
IL-17	GCTGACCCCTAAGAAACCCC	GAAGCAGTTTGGGACCCCTT
IL-6	GCCTACCGCCGATGAAGTTTCTCT	TATAATGCGGCCGCCTAGGTTTGCCGA
TNF-*α*	TCAGAATGAGGCTGGATAAGAT	GGAGCAGAGGTTCAGTGATGTA
Foxp3	CACCTATGCCACCCTTATCC	CGAACATGCGAGTAAACCAA
IL-10	ATGCTGCCTGCTCTTACTGACTG	CCCAAGTAACCCTTAAAGTCCTGC
GAPDH	AGCAATGCCTCCTGCACCACCAAC	CCGGAGGGGCCATCCACAGTCT

**Table 2:** Lipid profile of serum and body weight of ApoE-/- and ApoE-/- IL-12p35-/- mice.

	ApoE**-/-**			ApoE**-/-** IL-12p35**-/-**		
	8 weeks	18 weeks	24 weeks	8 weeks	18 weeks	24 weeks
TC (md/dl)	413 ± 122	928 ± 244	1047 ± 367	394 ± 135	887 ± 264	998 ± 402
TG (md/dl)	61 ± 33	88 ± 35	102 ± 49	53 ± 37	82 ± 35	94 ± 42
LDL-C (md/dl)	221 ± 54	924 ± 274	1074 ± 477	204 ± 48	894 ± 258	1184 ± 439
HDL-C (md/dl)	98 ± 27	64 ± 26	53 ± 22	109 ± 34	71 ± 30	58 ± 35
BD (g)	22.4 ± 1.0	26.7 ± 2.8	29.3 ± 5.4	21.8 ± 0.9	27.4 ± 3.3	29.7 ± 6.1

TC: total cholesterol; TG: total triglycerides; LDL-C: low-density lipoprotein cholesterol; HDL-C: high-density lipoprotein cholesterol; BD: body weight.

**Table 3:** Lipid profile of serum and body weight of ApoE-/-+PBS and ApoE-/-+rIL-35 mice.

	ApoE**-/-**			ApoE**-/-**		
	8 weeks	18 weeks	24 weeks	8 weeks	18 weeks	24 weeks
TC (md/dl)	427 ± 131	955 ± 204	1104 ± 398	414 ± 99	918 ± 229	1058 ± 326
TG (md/dl)	64 ± 31	92 ± 30	112 ± 38	59 ± 30	89 ± 29	105 ± 32
LDL-C (md/dl)	238 ± 61	966 ± 227	1136 ± 442	224 ± 56	942 ± 267	1201 ± 336
HDL-C (md/dl)	102 ± 31	69 ± 29	51 ± 25	113 ± 36	75 ± 33	62 ± 37
BD (g)	21.9 ± 1.8	27.2 ± 3.2	30.5 ± 6.2	22.2 ± 1.6	28.2 ± 3.8	30.4 ± 7.3

## Data Availability

The data used to support the findings of this study are available from the corresponding author upon request.
